# Supplementary material for: Tyr198 is the Essential Autophosphorylation Site for STK16 Localization and Kinase Activity
Source: Int J Mol Sci. 2019 Sep 30;20(19):4852. doi: 10.3390/ijms20194852 (PMC6801969; doi:10.3390/ijms20194852)
Supplement: Supplementary file 1 [file ijms-20-04852-s001.pdf]

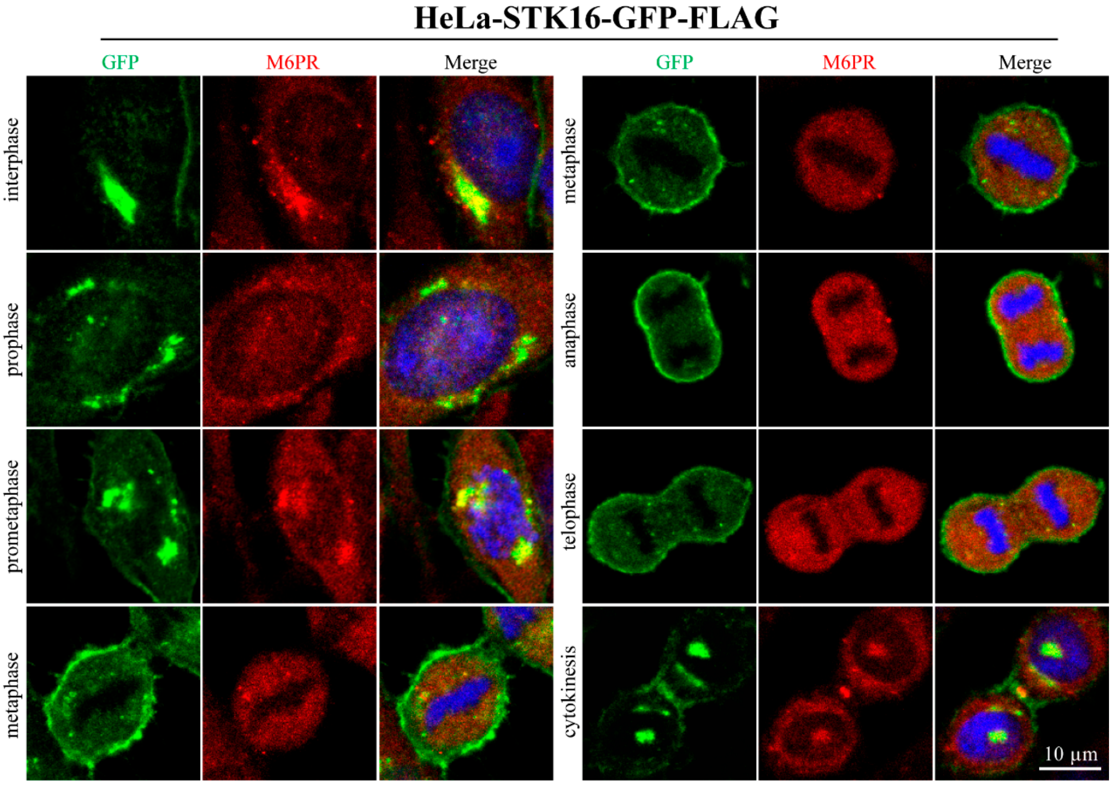

Figure S1. The subcellular localization of STK16 throughout the cell cycle. HeLa cells stably expressing STK16-GFP-FLAG were fixed and then subjected to anti-GFP antibody and anti-M6PR antibody staining. GFP staining of STK16 is shown in green, M6PR staining is shown in red, and DAPI staining of the nucleus is shown in blue. Experiments were repeated at least three times and representative results are shown. Scale bar, 10 μm.

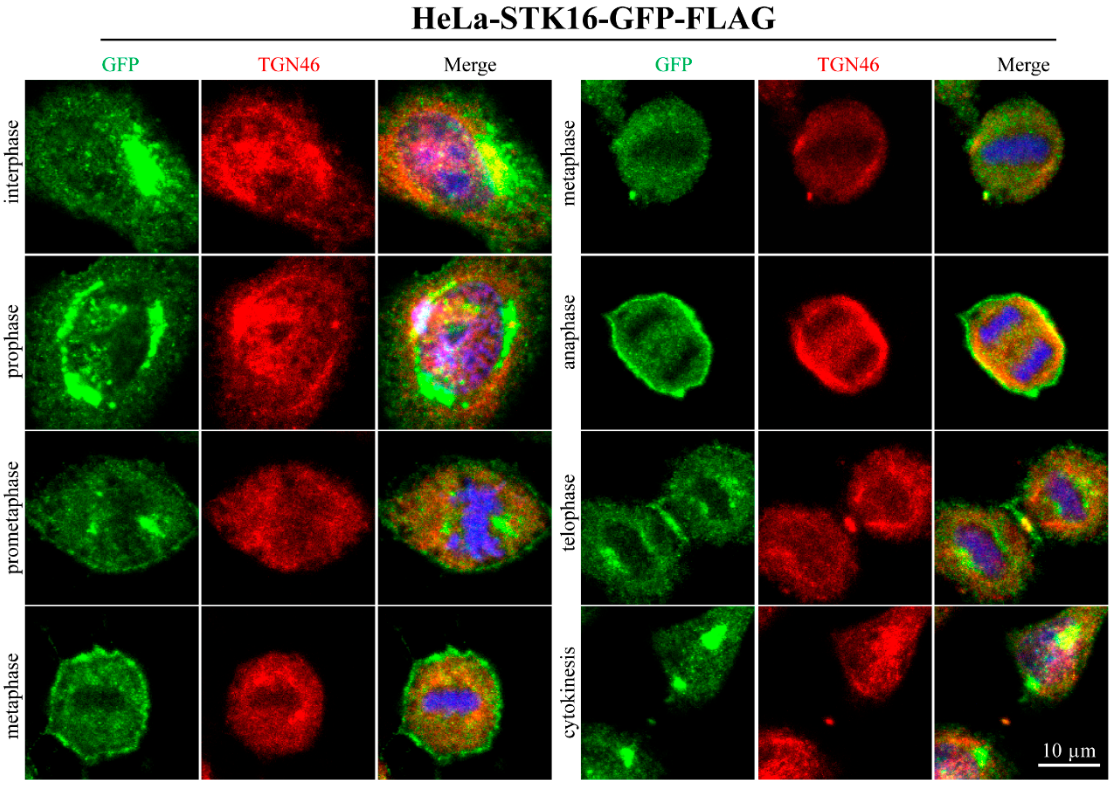

Figure S2. The subcellular localization of STK16 throughout the cell cycle. HeLa cells stably expressing STK16-GFP-FLAG were fixed and then subjected to anti-GFP antibody and anti-TGN46 antibody staining. GFP staining of STK16 is shown in green, TGN46 staining is shown in red, and DAPI staining of the nucleus is shown in blue. Experiments were repeated at least three times and representative results are shown. Scale bar, 10 μm.

Table S1. Oligonucleotides used to construct plasmids in this study.

Oligonucleotide	Sequence (5′-3′)
STK16-WT-GFP-FLAG FP	ACTGACCTCGAGGCCACCATGGGCCACGCGCTGTGTGTC
STK16-WT-GFP-FLAG RP	ACTGACGAATTCGATTTGGGTAGTATGTTGGCCAGGAGC
STK16-**T185E**-FMP	CAGGCTCTGGAACTGCAGGACTGGGCAGCCCAGC
STK16-**T185E**-RMP	GTCCTGCAGTTCCAGAGCCTGGCGGGAGCCCTC
STK16-**T185A**-FMP	CAGGCTCTGGCGCTGCAGGACTGGGCAGCCCAGC
STK16-**T185A**-RMP	GTCCTGCAGCGCCAGAGCCTGGCGGGAGCCCTC
STK16-**S197E**-FMP	GGCTCGGTATTCGATGGTGCACCGCTGGGCTGC
STK16-**S197E**-RMP	TGCACCATCGAATACCGAGCCCCAGAGCTCTTCT
STK16-**S197A**-FMP	GGCTCGGTAGGCGATGGTGCACCGCTGGGCTGC
STK16-**S197A**-RMP	TGCACCATCGCCTACCGAGCCCCAGAGCTCTTCT
STK16-**Y198E**-FMP	CTGGGGCTCGTTCGGAGATGGTGCACCGCTGGGCT
STK16-**Y198E**-RMP	CACCATCTCCGAACGAGCCCCAGAGCTCTTCTCTGTGC
STK16-**Y198A**-FMP	CTGGGGCTCGGGCGGAGATGGTGCACCGCTGGGCT
STK16-**Y198A**-RMP	CACCATCTCCGCCCGAGCCCCAGAGCTCTTCTCTGTGC
STK16-**S197E-Y198E**-FMP	TGGGGCTCGTTCTTCGATGGTGCACCGCTGGGCTGCCCA
STK16-**S197E-Y198E**-RMP	TGCACCATCGAAGAACGAGCCCCAGAGCTCTTCTCTGTGC
STK16-**S197A-Y198A**-FMP	TGGGGCTCGGGCAGCGATGGTGCACCGCTGGGCTGCCCA
STK16-**S197A-Y198A**-RMP	TGCACCATCGCTGCCCGAGCCCCAGAGCTCTTCTCTGTGC

Point mutations are indicated in bold type, and affected codon(s) are underlined and marked in red. FP, forward primer; RP, reverse primer; FMP, forward mutant primer; RMP, reverse mutant primer.

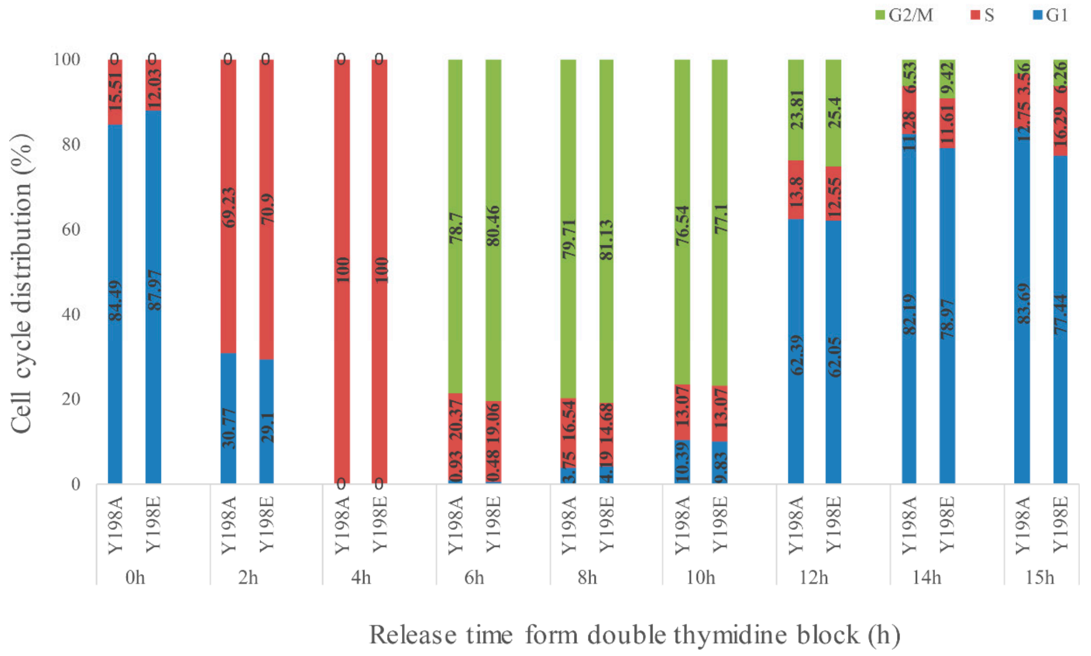

Figure S3. HeLa-STK16 Y198E-GFP-FLAG and HeLa-STK16 Y198A-GFP-FLAG cell lines were synchronized to the G1/S phase by double thymidine, and the cell cycle was detected by FACS at the times indicated.
